# Investigation of the Propagation of Stress Wave in Nickel-Titanium Shape Memory Alloys

**DOI:** 10.3390/ma11071215

**Published:** 2018-07-15

**Authors:** Yehui Cui, Xiangguo Zeng, Huayan Chen, Jun Chen, Fang Wang

**Affiliations:** 1 College of Architecture and Environment, Sichuan University, Chengdu 610065, China; 2016223050020@stu.scu.edu.cn (Y.C.); huayanchen@scu.edu.cn (H.C.); 2 Laboratory of Computational Physics, Institute of Applied Physics and Computational Mathematics, Beijing 100088, China; jun_chen@iapcm.ac.cn; 3 Center for Applied Physics and Technology, Peking University, Beijing 100071, China; 4 Faculty of Materials and Energy, Southwest University, Chongqing 400715, China

**Keywords:** constitutive model, NiTi alloys, stress wave propagation, material point method

## Abstract

Based on irreversible thermodynamic theory, a new constitutive model incorporating two internal variables was proposed to investigate the phase transformation and plasticity behavior in nickel-titanium (NiTi) shape memory alloys (SMAs), by taking into account four deformation stages, namely austenite elastic phase, phase transition, martensitic elastic phase, and plastic phase. The model using the material point method (MPM) was implemented by the FORTRAN code to investigate the stress wave and its propagation in a NiTi rod. The results showed that its wave propagation exhibited martensitic and austenitic elastic wave, phase transition wave, and plastic wave. However, a double-wave structure including the martensitic and austenitic elastic wave and plastic wave occurred when the martensitic elastic wave reached the phase transformation wave. Thus, the reflection wave at a fixed boundary exhibited a different behavior compared with the elastic one, which was attributed to the phase transition during the process of reflection. It was found that the stress increment was proportional to the velocity of phase transition wave after the stress wave reflection. In addition, the influences of loading direction and strain rate on the wave propagation were examined as well. It was found that the phase transition wave velocity increased as the strain rate increased. The elastic wave velocity of martensite under compressive conditions was larger than that under tensile loading. In contrast, the plastic wave velocity under compression was less than that subjected to the tensile load.

## 1. Introduction

Due to an escalating growth of advanced technologies, shape memory alloys (SMAs) have been used in a wide variety of fields involving medical, aeronautical, and automotive because of two remarkable properties: superelasticity and shape memory effect [1]. The origin of these properties is characterized by the martensitic transition (MT) and its reverse (austenitic transition (AT)) occurring in such materials. Indeed, martensite (M) stabilizes at low temperature and high stress, and conversely austenite (A) stabilizes at high temperature and low stress [2]. Due to functional properties as well as high strength and ductility, a nickel-titanium (NiTi) alloy has been considered as one of the most promising alloys.

In recent decades, much work has been done on the development of a constitutive model of SMAs to describe unique mechanical behaviors, as reviewed by Cisse et al. [3]. These models are mainly divided into two groups: one is the micromechanical model, and the other is the macro-phenomenological model. Manchiraju et al. [4] developed a model based on the finite element method to study the interaction between martensitic transformation and plasticity in NiTi SMAs from the point of view of microstructure. Yu et al. [5,6] also performed some investigations on the thermo-mechanical and anisotropic deformation behavior of superelastic NiTi alloys. It is justified from these studies that molecular dynamics simulations could be deemed as an effective approach for investigation the MT and AT by providing more structural details at the atomic scale. However, the choice of interatomic potential has a significant influence on simulations. Ackland et al. [7] used the embedded-atom method potential to simulate the MT in NiTi. They found that the phase transition was accompanied by the instability of precursor long wave length and rotation of the matrix. Kastner et al. [8] simulated the microstructure transformation behavior during cyclic loading using the Lennard-Jones potential. The results showed that the accumulation of permanent damage in the martensite led to functional fatigue.

The advantage of the micromechanics-based constitutive model is that it could predict the structural response from the view of physical nature. However, it cannot meet the need of engineering application due to computational complexities. Thus, the phenomenological constitutive model is more suitable for engineering. Indeed, the work of developing the phenomenological constitutive equations for SMA was divided into the two following groups:

### 1.1. Thermodynamic

Tanaka et al. [9] first developed the one-dimensional model using internal variables under the framework of thermomechanics. Boyd and Lagoudas [10,11] also proposed some models that accounted for martensite reorientation by using a free energy function and a dissipation potential, respectively. Based on the above-mentioned models, Lagoudas et al. [12] established a new model that characterized three response stages of SMAs, which had not been solved with a unified manner by previous studies.

### 1.2. Generalized Plasticity

Auricchio et al. [13] proposed a model for investigating the superelastic behavior of SMAs under the framework of the generalized plasticity work. Considering the loading path of Durcker-Prgaer, the transformation from austenite to martensite and its reverse process were studied comprehensively. Interestingly, the martensite content and its variation during the phase transition were taken as two independent internal variables. Auricchio [14] gave the exponential and linear form for describing martensite evolution, respectively. The results demonstrated that the proposed model was effective for the isothermal loading/unloading description of the superelastic SMAs. Furthermore, Kan and Kang [15] proposed a model that accounted for the evolutions of residual induced-martensite and transformation-induced plastic strain under the stress-controlled cyclic loading. In our recent work, a phenomenological constitutive model was proposed by using irreversible thermodynamics with a semi-implicit stress integration algorithm [16]. Two internal variables were taken to describe the irreversible processes of phase transformation and dislocation evolution of NiTi alloys, where one variable represented the phase transition behavior, and the other denoted the plastic behavior.

An investigation on the wave propagation is one of the most important tasks in the field of material and computational mechanics. Sadeghi et al. [17] conducted the experiments and performed finite element calculations for energy dissipation during the phase transformation in NiTi alloys. However, the proposed constitutive model was verified to be inconsistent with the experiment results. Wang et al. [18,19] studied the dynamic deformation in NiTi SMAs at high strain rates of 10^6^~10^7^/s by laser. They found that there had a critical peak pressure for the NiTi alloy to induce martensitic transformation at higher strain rate. Bekker et al. [20] employed a thermodynamic-based constitutive model to investigate the propagation of phase transformation wave in SMAs. Furthermore, Fǎciu et al. [21] investigated the Goursat and Riemann problems of phase material dynamics by considering a piecewise linear-elastic function. Unfortunately, little work has been done in terms of studying the phase transformation and plastic wave propagation of SMAs.

Since there are some problems in the traditional finite element method (FEM), such as mesh distortion and element entanglement, the material point method (MPM) has been widely used to simulate material extreme deformation and failure. For example, Dong et al. [22] took an investigation on the effect of the impact forces on pipeline during the submarine landslide. Liu et al. [23] investigated the micron particles impact with high-velocity by using MPM, and the simulation results agreed well with the experimental ones. Also, MPM was extended to simulate the explosively driven response of metals by Lian et al. [24] and found that the MPM results were in reasonable agreement with the Gurney solutions. It is worth mentioning that there is a remarkable difference between FEM and MPM. In other words, FEM is a pure Lagrangian method, while MPM takes a material as considerable Lagrangian particles that move through a background in Eulerian mesh [25].

Due to advantages of MPM, this work attempts to develop an irreversible thermodynamic constitutive model based on MPM to simulate the wave propagations that represent phase transformation and plastic behavior. The wave structures are investigated with the help of MPM. The influences of loading direction and strain rate on the wave propagation are examined as well. It is worth mentioning that FORTRAN is employed as the programming language to perform the numerical calculation in this study.

## 2. Theoretical Framework

### 2.1. Thermodynamics

According to irreversible thermodynamics, it is assumed that the Helmholtz free energy function of a material point has a following expression

(1) $$\psi =\psi (\varepsilon_{ij}^{e},T,\eta ,\xi ),$$

where $\varepsilon_{ij}^{e}$ is the elastic strain tensor, and *T* is the temperature. *η*, *ξ* is two internal variables that characterize the plastic behavior and phase transition, respectively.

In the thermodynamics, the dissipation (*D*) of a unit is required to be positive (*D* ≥ 0) to ensure that the rate of stored energy is always larger than the stress power. Thus, the inequality of dissipation is obtained according to the Clausius-Duhem form

(2) $$D=\sigma_{ij}:\;{\dot{\varepsilon }}_{ij}-\rho \dot{\psi }\geq 0,$$

where $\varepsilon_{ij}$ denotes the total strain that is decomposed into the elastic strain and the inelastic strain. *ρ* denotes the material density. Besides, the total inelastic strain consists of two parts: one is the phase transition strain $\varepsilon_{ij}^{tr}$, and the other is the plastic strain $\varepsilon_{ij}^{p}$

(3) $$\varepsilon_{ij}=\varepsilon_{ij}^{e}+\varepsilon_{ij}^{in}=\varepsilon_{ij}^{e}+\varepsilon_{ij}^{tr}+\varepsilon_{ij}^{p},$$

Under isothermal conditions, if taking the time derivative of *ψ* in Equation (1), the following equation is obtained

(4) $$\dot{\psi }=\frac{\partial \psi }{\partial \varepsilon_{ij}^{e}}{\dot{\varepsilon }}_{ij}^{e}+\frac{\partial \psi }{\partial \eta }\dot{\eta }+\frac{\partial \psi }{\partial \xi }\dot{\xi },$$

Substituting Equations (3) and (4) into Equation (2), the dissipation inequality states

(5) $$(\sigma_{ij}-\rho \frac{\partial \psi }{\partial \varepsilon_{ij}^{e}}){\dot{\varepsilon }}_{ij}^{e}+(\sigma_{ij}{\dot{\varepsilon }}_{ij}^{tr}-\rho \frac{\partial \psi }{\partial \xi }\dot{\xi })+(\sigma_{ij}{\dot{\varepsilon }}_{ij}^{p}-\rho \frac{\partial \psi }{\partial \eta }\dot{\eta })\geq 0,$$

Due to an assumption that the elastic strain, phase transition strain and plastic strain are independent, internal variables result in the dissipation of free energy. The dissipation inequality, shown as Equation (5), requires that internal variables should satisfy the following equations

(6a) $$\sigma_{ij}=\rho \frac{\partial \psi }{\partial \varepsilon_{ij}^{e}},$$

(6b) $$\sigma_{ij}{\dot{\varepsilon }}_{ij}^{tr}-\rho \frac{\partial \psi }{\partial \xi }\dot{\xi }\geq 0,$$

(6c) $$\sigma_{ij}{\dot{\varepsilon }}_{ij}^{p}-\rho \frac{\partial \psi }{\partial \eta }\dot{\eta }\geq 0,$$

Indeed, these represent the elastic, phase transition and plastic evolution, respectively.

### 2.2. Governing Equations

Similar to *ε_ij_*, *ψ* is decomposed into the elastic free energy *ψ_e_*, the phase transition free energy *ψ_tr_*, and the plastic free energy *ψ_p_*

(7) $$\psi =\psi_{e}+\psi_{tr}+\psi_{p},$$

where *ψ_e_* at a material point needs to satisfy the following equation

(8) $$\rho \psi_{e}=\frac{1}{2}(2\mu \varepsilon_{ij}^{e}\varepsilon_{ij}^{e}+\lambda \varepsilon_{kk}^{e}\varepsilon_{kk}^{e}),$$

where *λ* and *μ* are Lamé parameters.

The substitution of Equations (7) and (8) into Equation (6a) produces

(9a) $$\sigma_{ij}=2\mu \varepsilon_{ij}^{e}+\lambda \varepsilon_{kk}^{e}\delta_{ij},$$

Equation (9a) that represents Hook’s law is expressed as another form

(9b) $$\sigma_{ij}=E_{ijkl}\varepsilon_{kl}^{e}=E_{ijkl}(\varepsilon_{kl}-\varepsilon_{kl}^{tr}-\varepsilon_{kl}^{p}),$$

While SMAs contain martensite and austenite phases, an equivalent elastic stiffness matrix $E_{ijkl}$ is a function of the martensitic volume fraction *n*, which can be determined by the simple Voigt form [12]

(10) $$E_{ijkl}(n)=(1-n)E_{ijkl}^{A}+nE_{ijkl}^{M},$$

### 2.3. Phase Transition Evolution

Under the framework of the thermodynamics, the evolution of phase transition needs to satisfy Equation (6b).

Assuming that

(11) $$A=-\rho \frac{\partial \psi }{\partial \xi },$$

where *A* is a generalized force that is dependent on the internal variable *ξ*, and then Equation (6b) is updated as

(12) $$\sigma_{ij}{\dot{\varepsilon }}_{ij}^{tr}+A\dot{\xi }\geq 0,$$

A potential function is introduced

(13) $$\Theta =\Theta (\sigma_{ij},A),$$

Then, the evolution equations are expressed as

(14a) $${\dot{\varepsilon }}_{ij}^{tr}={\dot{\lambda }}_{1}\frac{\partial \Theta }{\partial \sigma_{ij}},$$

(14b) $$\dot{\xi }={\dot{\lambda }}_{1}\frac{\partial \Theta }{\partial A},$$

Substituting Equation (14) into Equation (12), the inequality also states

(15) $${\dot{\lambda }}_{1}(\sigma_{ij}\frac{\partial \Theta }{\partial \sigma_{ij}}+A\frac{\partial \Theta }{\partial A})\geq 0,$$

Two conditions are required to satisfy Equation (14b), i.e., one is that the potential function $\Theta =\Theta \left(\sigma_{ij},A\right)$, with respect to $\sigma_{ij}$ and *A*, is convex outward. The other is that ${\dot{\lambda }}_{1}$ cannot be negative. If the Helmholtz energy of phase transition has the form of

(16) $$\rho \psi_{tr}=\frac{1}{2}k_{1}\xi_{ij}\xi_{ij},$$

A potential function $\Theta =\Theta \left(\sigma_{ij},A\right)$ that is analogous to the classical Chaboche plastic constitutive model [26] is proposed as follows

(17) $$\Theta =\Theta (\sigma_{ij},A)={\left[\frac{3}{2}(s_{ij}-A_{ij}^{\prime })(s_{ij}-A_{ij}^{\prime })\right]}^{\frac{1}{2}}+\frac{1}{2}aA_{ij}A_{ij},$$

where $s_{ij}$ is the stress partial tensor, and *A*′ is the deviator of the generalized force $A_{ij}$. *a* is an arbitrary value when constructing the function.

${\dot{\lambda }}_{1}$ is taken as the form of

(18) $${\dot{\lambda }}_{1}={\left(\frac{F_{y}^{tr}}{Z_{1}}\right)}^{n_{1}},$$

where *Z*_1_ is a material parameter. $F_{y}^{tr}$ represents the phase transition yield surface, as follows

(19) $$F_{y}^{tr}=\sigma_{eq}-\sigma_{s}^{tr}(n)=\sqrt{\frac{3}{2}(s_{ij}-A_{ij}^{\prime }):(s_{ij}-A_{ij}^{\prime })}-\sigma_{s}^{tr}(n),$$

where *n* is Martensitic volume fraction, which is defined as

(20) $$n=\frac{\varepsilon_{eq}^{tr}}{\varepsilon_{m}},$$

where $\varepsilon_{eq}^{tr}=\sqrt{\frac{2}{3}\varepsilon_{ij}^{tr}:\varepsilon_{ij}^{tr}}$ is the equivalent strain of phase transition, and *ε_m_* is the maximum strain of phase transition under uniaxial loading. The relationship of initial phase transition stress $\sigma_{s}^{tr}$ and the final one $\sigma_{f}^{tr}$ is expressed as follows

(21) $$\sigma_{s}^{tr}(n)=(1-n)\sigma_{s}^{tr}+n\sigma_{f}^{tr},$$

where $\sigma_{s}^{tr}$ and $\sigma_{f}^{tr}$ are related to the temperature and the strain rate, respectively.

Hence, the following equation could be used to describe the behavior that is dependent on the temperature and the strain rate

(22a) $$\sigma_{s}^{tr}=\sigma_{s0}^{tr}[1+C_{1}\ln(\frac{\dot{\varepsilon }}{{\dot{\varepsilon }}_{0}})][1+m_{1}(T-T_{0})],$$

(22b) $$\sigma_{f}^{tr}=\sigma_{f0}^{tr}[1+C_{2}\frac{\dot{\varepsilon }}{{\dot{\varepsilon }}_{0}}][1+m_{2}(T-T_{0})],$$

Substituting Equations (11)–(20) into Equations (14) and (15), the evolution of phase transition of NiTi SMAs is provided as the following form

(23a) $${\dot{\varepsilon }}_{ij}^{tr}={\dot{\lambda }}_{1}\frac{\partial \Theta }{\partial \sigma_{ij}}=\frac{3}{2}{\left(\frac{F_{y}^{tr}}{Z_{1}}\right)}^{n_{1}}\frac{s_{ij}-A_{ij}^{\prime }}{\sigma_{eq}^{tr}},$$

(23b) $${\dot{A}}_{ij}=k_{1}{\dot{\varepsilon }}_{ij}^{tr}-k_{2}{\dot{\varepsilon }}_{eq}^{tr}A_{ij},$$

### 2.4. Evolution of Plasticity

Similarly, the evolution of phase transitions should satisfy Equation (6c).

Assuming that

(24) $$B=-\rho \frac{\partial \psi }{\partial \eta },$$

where *B* is a generalized force that is related to the internal variable *η*. Then, Equation (6c) is updated as follows

(25) $$\sigma_{ij}{\dot{\varepsilon }}_{ij}^{p}+B\dot{\eta }\geq 0,$$

Assuming a potential function

(26) $$\Omega =\Omega (\sigma_{ij},B),$$

Consequently, the evolution equations are expressed as follows

(27a) $${\dot{\varepsilon }}_{ij}^{p}={\dot{\lambda }}_{2}\frac{\partial \Omega }{\partial \sigma_{ij}},$$

(27b) $$\dot{\eta }={\dot{\lambda }}_{2}\frac{\partial \Omega }{\partial B},$$

The substitution of Equation (27a,b) into Equation (25) produces the following inequality

(28) $${\dot{\lambda }}_{2}(\sigma_{ij}\frac{\partial \Omega }{\partial \sigma_{ij}}+B\frac{\partial \Omega }{\partial B})\geq 0,$$

Two conditions are needed to satisfy Equation (25), i.e., one is the potential function $\Omega =\Omega \left(\sigma_{ij},B\right)$ with respect to variables, which is convex outward. The other is that ${\dot{\lambda }}_{2}$ are not be negative.

Assuming the Helmholtz energy of phase transition has the form of

(29) $$\rho \psi_{p}=\frac{1}{2}k_{2}\eta_{ij}\eta_{ij},$$

A potential function $\Omega =\Omega \left(\sigma_{ij},B\right)$ that is analogous to the classical Chaboche plastic constitutive model [26] is provided as follows

(30) $$\Omega =\Omega (\sigma_{ij},B)={\left[\frac{3}{2}(s_{ij}-B_{ij}^{\prime })(s_{ij}-B_{ij}^{\prime })\right]}^{\frac{1}{2}}+\frac{1}{2}bB_{ij}B_{ij},$$

where *S_ij_* is the stress partial tensor, and $B_{ij}^{\prime }$ is the deviator of the generalized force $B_{ij}$. *b* is an arbitrary value when constructing the function.

If ${\dot{\lambda }}_{2}$ has the form of

(31) $${\dot{\lambda }}_{2}={\left(\frac{F_{y}^{p}}{Z_{2}}\right)}^{n_{2}},$$

The plastic yield surface $F_{y}^{p}$ is written as

(32) $$F_{y}^{p}=\sigma_{eq}^{p}-\sigma_{y}^{p}-R=\sqrt{\frac{3}{2}(s_{ij}-B_{ij}^{\prime }):(s_{ij}-B_{ij}^{\prime })}-\sigma_{y}^{p}-R,$$

where $\sigma_{y}^{p}$ represents the initial plastic yield stress. The isotropic hardening phenomenon is described by *R* that is defined as

(33) $$\dot{R}=m(R_{1}-R){\dot{\varepsilon }}_{eq}^{p},$$

where ${\dot{\varepsilon }}_{eq}^{p}=\sqrt{\frac{2}{3}{\dot{\varepsilon }}^{p}:{\dot{\varepsilon }}^{p}}$ is the equivalent plastic strain increment, and *m* and *R*_1_ are the material parameters for characterizing the plastic hardening behavior.

Therefore, the evolution of plastic of NiTi SMAs has the following form

(34a) $${\dot{\varepsilon }}_{ij}^{p}={\dot{\lambda }}_{2}\frac{\partial \Omega }{\partial \sigma_{ij}}=\frac{3}{2}{\left(\frac{F_{y}^{p}}{Z_{2}}\right)}^{n_{2}}\frac{s_{ij}-B_{ij}^{\prime }}{\sigma_{eq}^{p}},$$

(34b) $${\dot{B}}_{ij}=k_{3}{\dot{\varepsilon }}_{ij}^{p}-k_{4}{\dot{\varepsilon }}_{eq}^{p}B_{ij},$$

### 2.5. Iteration Algorithm

**Figure 1:**
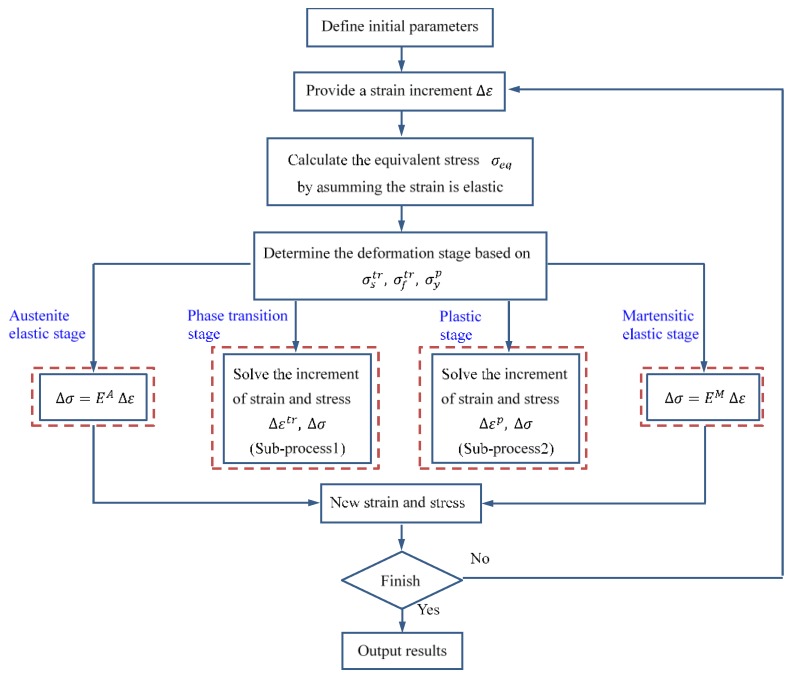
Flow chart of the iteration solution.

Figure 1 shows a flow chart of the iteration solution procedure. In this algorithm, the semi-implicit stress integration method is employed to solve the constitutive model equivalent inelastic in the constitutive model. The procedure is outlined as follows: first, the initial values (such as stress, time step) are defined and all strains are assumed to be elastic. Second, the stress is calculated to determine the deformation stage.

**Figure 2:**
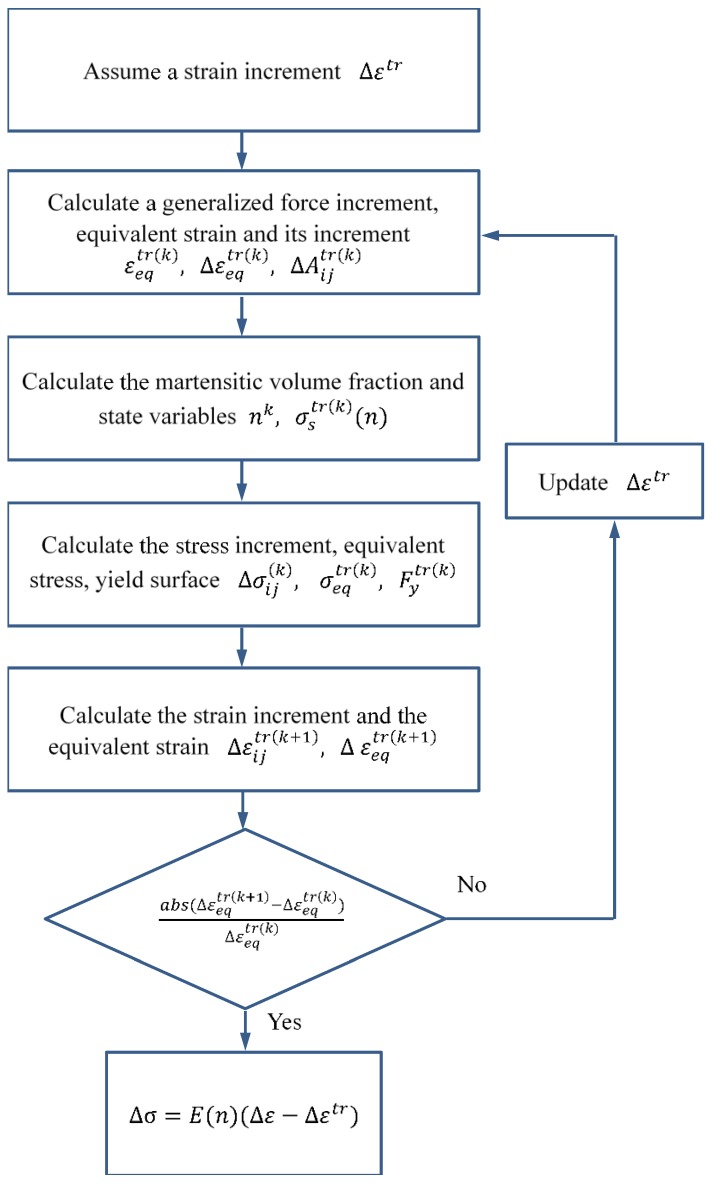
Flow chart of the solution of phase transition stage.

**Figure 3:**
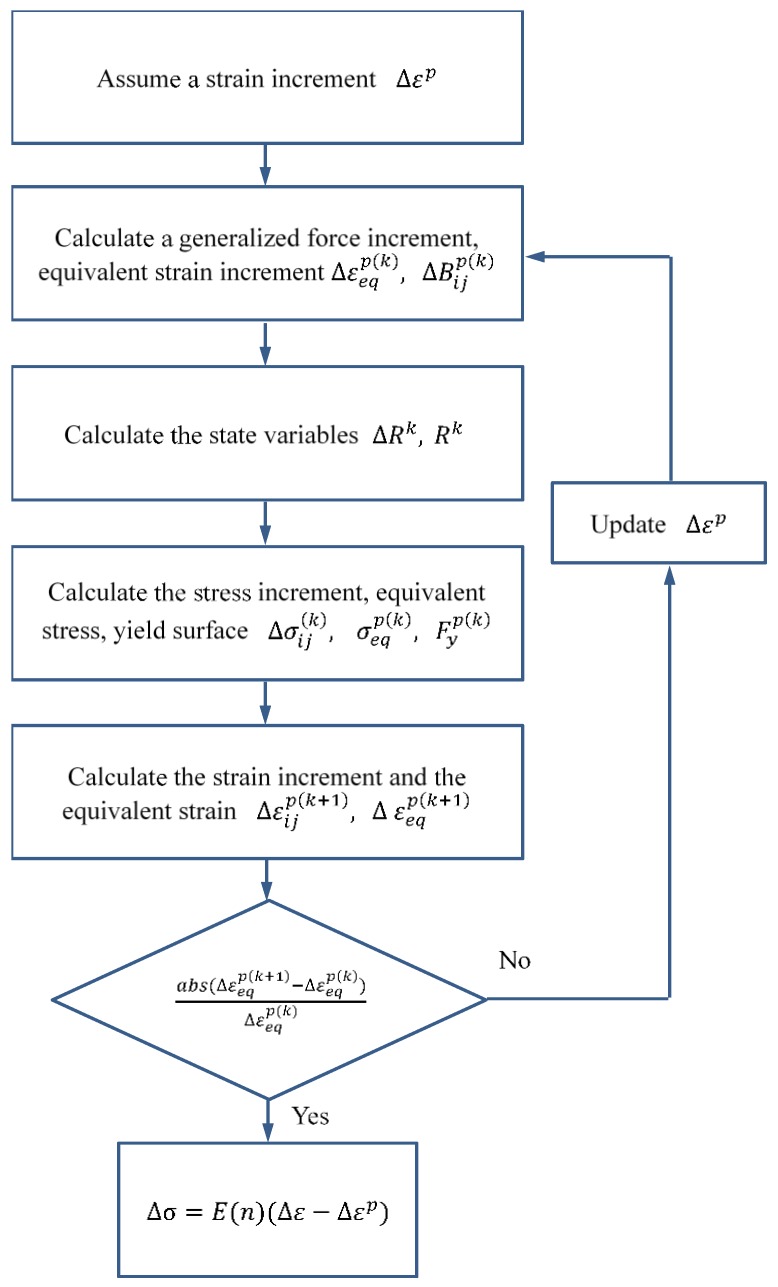
Flow chart of the solution of plastic stage.

In addition, the flow chart for solving the phase transition stage and plastic stage are provided in Figure 2 and Figure 3, respectively.

The solution of the stress–strain response during the phase transition stage and plastic stage is outlined below.

*Step 1.* An inelastic strain increment is assumed to be

(35) $$\Delta \varepsilon_{ij}^{in^{\left(0\right)}}=\delta_{0},$$

*Step 2*. The generalized force is calculated, and then the equivalent inelastic strain and its increment is derived from:

Phase transition stage:

(36a) $$\varepsilon_{eq}^{tr^{\left(k\right)}}=\sqrt{\frac{2}{3}\varepsilon_{ij}^{tr^{\left(k\right)}}\varepsilon_{ij}^{tr^{\left(k\right)}}},$$

(36b) $$\Delta \varepsilon_{eq}^{tr^{\left(k\right)}}=\sqrt{\frac{2}{3}\Delta \varepsilon_{ij}^{tr^{\left(k\right)}}\Delta \varepsilon_{ij}^{tr^{\left(k\right)}}},$$

(36c) $$\Delta A_{ij}^{\left(k\right)}=k_{1}\Delta \varepsilon_{ij}^{tr^{\left(k\right)}}-k_{2}\Delta \varepsilon_{eq}^{tr^{\left(k\right)}}A_{ij}^{\left(k\right)},$$

Plastic stage:

(37a) $$\varepsilon_{eq}^{p^{\left(k\right)}}=\sqrt{\frac{2}{3}\varepsilon_{ij}^{p^{\left(k\right)}}\varepsilon_{ij}^{p^{\left(k\right)}}},$$

(37b) $$\Delta \varepsilon_{eq}^{p^{\left(k\right)}}=\sqrt{\frac{2}{3}\Delta \varepsilon_{ij}^{p^{\left(k\right)}}\Delta \varepsilon_{ij}^{p^{\left(k\right)}}},$$

(37c) $$\Delta B_{ij}^{\left(k\right)}=k_{3}\Delta \varepsilon_{ij}^{p^{\left(k\right)}}-k_{4}\Delta \varepsilon_{eq}^{p^{\left(k\right)}}B_{ij}^{\left(k\right)},$$

*Step 3.* Accordingly, the state variables are determined:

Phase transition stage:

(38a) $$n^{\left(k\right)}=\frac{\varepsilon_{eq}^{tr(k)}}{\varepsilon_{m}},$$

(38b) $$\sigma_{s}^{tr(k)}(n)=(1-n^{\left(k\right)})\sigma_{s}^{tr}+n^{\left(k\right)}\sigma_{f}^{tr},$$

Transition stage:

(39) $$\Delta R^{\left(k\right)}=m(R_{1}-R^{\left(k\right)})\Delta \varepsilon_{eq}^{p(k)},$$

*Step 4*. The corresponding stress increment is calculated

(40a) $$E_{ijkl}^{\left(k\right)}(n)=(1-n^{\left(k\right)})E_{ijkl}^{A}+n^{\left(k\right)}E_{ijkl}^{M},$$

(40b) $$\Delta \sigma_{ij}^{\left(k\right)}=E_{ijkl}^{\left(k\right)}(\Delta \varepsilon_{ij}-\Delta \varepsilon_{ij}^{in(k)}),$$

*Step 5*. Generalized force and stress are updated as

(41a) $$A_{ij}^{\left(k+1\right)}=A_{ij}^{\left(k\right)}+\Delta A_{ij}^{\left(k\right)},$$

(41b) $$B_{ij}^{\left(k+1\right)}=B_{ij}^{\left(k\right)}+\Delta B_{ij}^{\left(k\right)},$$

(41c) $$\sigma_{ij}^{\left(k+1\right)}=\sigma_{ij}^{\left(k\right)}+\Delta \sigma_{ij}^{\left(k\right)},$$

*Step 6.* In turn, the equivalent stress and the yield surface are solved.

Phase transition stage:

(42a) $$\sigma_{eq}^{tr(k)}=\sqrt{\frac{3}{2}(s_{ij}^{\left(k\right)}-A_{ij}^{{}^{\prime }(k)}):(s_{ij}^{\left(k\right)}-A_{ij}^{{}^{\prime }(k)})},$$

(42b) $$F_{y}^{tr(k)}=\sigma_{eq}^{tr(k)}-\sigma_{y}^{tr(k)},$$

Plastic stage:

(43a) $$\sigma_{eq}^{p(k)}=\sqrt{\frac{3}{2}(s_{ij}^{\left(k\right)}-B_{ij}^{{}^{\prime }(k)}):(s_{ij}^{\left(k\right)}-B_{ij}^{{}^{\prime }(k)})},$$

(43b) $$F_{y}^{p(k)}=\sigma_{eq}^{p(k)}-\sigma_{y}^{p(k)},$$

*Step 7*. The new equivalent inelastic strain increment is as follows.

Phase transition stage:

(44a) $$\Delta \varepsilon_{ij}^{tr(k+1)}=\frac{3}{2}{\left(\frac{F_{y}^{tr(k)}}{Z_{1}}\right)}^{n_{1}}\frac{s_{ij}^{\left(k\right)}-A_{ij}^{\left(k\right)}}{\sigma_{eq}^{tr(k)}}\Delta t,$$

(44b) $$\Delta \varepsilon_{eq}^{tr(k+1)}=\sqrt{\frac{2}{3}\Delta \varepsilon_{ij}^{tr(k)}\Delta \varepsilon_{ij}^{tr(k)}},$$

Plastic stage:

(45a) $$\Delta \varepsilon_{ij}^{p(k+1)}=\frac{3}{2}{\left(\frac{F_{y}^{p(k)}}{Z_{2}}\right)}^{n_{2}}\frac{s_{ij}^{\left(k\right)}-B_{ij}^{\left(k\right)}}{\sigma_{eq}^{p(k)}}\Delta t,$$

(45b) $$\Delta \varepsilon_{eq}^{p(k+1)}=\sqrt{\frac{2}{3}\Delta \varepsilon_{ij}^{p(k)}\Delta \varepsilon_{ij}^{p(k)}},$$

*Step 8*. The convergence of iteration is checked:

(46) $$\frac{abs(\Delta \varepsilon_{eq}^{in^{\left(k+1\right)}}-\Delta \varepsilon_{eq}^{in^{\left(k\right)}})}{\Delta \varepsilon_{eq}^{in^{\left(k\right)}}}<err,$$

A dynamic compression experiment was carried out, and the alloy was made into a cylinder of *ϕ* 8 × 4 mm. Material chemical compositions are provided in Table 1. Table 2 and Table 3 provide material parameters under compressive and tensile loading, respectively.

**Figure 4:**
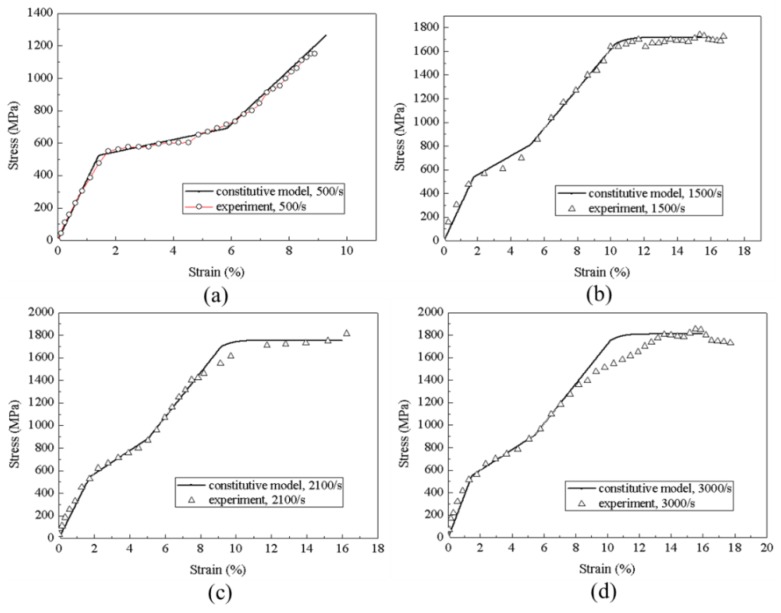
Dependence of the stress–strain response on various strain rates of (**a**) 500/s; (**b**) 1500/s; (**c**) 2100/s; (**d**) 3000/s.

Figure 4 provides a comparison between the calculated values and experimental results, which shows that the predicted results are in reasonable agreement with experimental data, highlighting the practicability of the proposed model for describing such a behavior.

### 2.6. MPM

**Figure 5:**
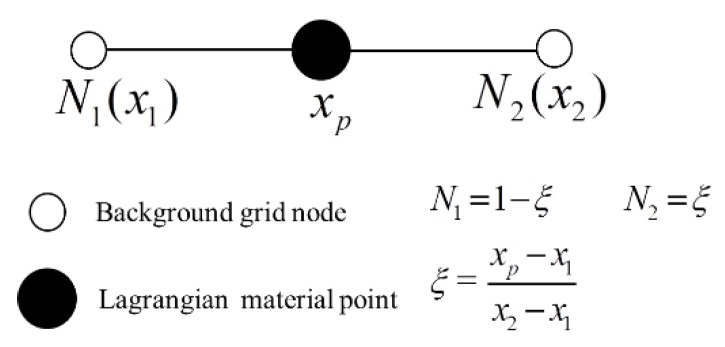
A shape function for connecting material point with background grid node *s*.

Similar to the concept of FEM, background grids and material points with physical meaning (such as mass, stress, strain) are meshed firstly in MPM. As mentioned in the introduction, these background grids are the Eulerian meshes, and it remains fixed during the process of calculation. A material point could be connected with a background grid by using a shape function, shown in Figure 5.

In each time *t*, the variables of Lagrangian material points are mapped into the Eulerian grid nodes by the shape function

(47) $$G_{i}^{t}=\sum_{p=1}^{N_{p}}G_{p}^{t}N_{i}(x_{p}^{t}),$$

where $N_{i}\left(x_{p}^{t}\right)$ is the shape function of a material point *p* in each time step. The subscript *i* denotes the node number of background grid. $G_{i}^{t}$ and $G_{p}^{t}$ represent the grid nodes and the material point at *t*, respectively. In a similar way, the meaning is extended to the mass $\left(m_{i}^{t},m_{p}^{t}\right)$, the coordinate $\left(x_{i}^{t},x_{p}^{t}\right)$, the displacement $\left(u_{i}^{t},u_{p}^{t}\right)$, the velocity $\left(v_{i}^{t},v_{p}^{t}\right)$, and the acceleration $\left(a_{i}^{t},a_{p}^{t}\right)$.

The external force at each material point includes an external load and a volume force, which is expressed as

(48) $$c_{i}^{t}=\sum_{p=1}^{N_{p}}m_{p}^{t}c_{p}^{s,t}h^{-1}N_{i}(x_{p}^{t}),$$

where $c_{p}^{s,t}$ is the external load of *p* at *t*. *h* is the number of cell layers applied to the material point.

The material point volume force is used to map the volume force of the background node

(49) $$b_{i}^{t}=\sum_{p=1}^{N_{p}}m_{p}^{t}b_{p}^{s,t}N_{i}(x_{p}^{t}),$$

where $b_{p}^{s,t}$ is the volume force of *p* at *t*.

The external force of a node is determined by a combination of Equations (48) and (49)

(50) $${\left(f_{i}^{t}\right)}^{ext}=c_{i}^{t}+b_{i}^{t},$$

Afterwards, the internal force on the material point is mapped to the background grid nodes

(51) $${\left(f_{i}^{t}\right)}^{\mathrm{int}}=-\sum_{p=1}^{N_{p}}m_{p}^{t}S_{p}^{s,t}\nabla N_{i}(x_{p}^{t}),$$

where $S_{p}^{s,t}$ is the Cauchy stress of *p* at *t*.

Then, the background node mass, node speed, internal force and external force of node are achieved through the mapping calculation of the results.

Indeed, the model of impact behavior is mainly governed by equations of mass conservation and momentum conservation.

Mass conservation:

(52) $$\frac{d\rho (x,t)}{dt}+\rho (x,t)\nabla v=0,$$

Momentum conservation:

(53) ρ(x,t)a=∇s+ρ(x,t)b,

where ρ(x,t) is the density. *v* is the velocity. *a* is the acceleration. *s* is the Cauchy stress. The density is expressed as follows

(54) $$\rho_{p}^{t}=\sum_{p=1}^{N_{p}}m_{p}^{t}\delta (x-x_{p}^{t}),$$

Taking the test function as *w*, the weak form of momentum equation is obtained as follows

(55) $$\int_{\Omega }\rho w\cdot ad\Omega =-\int_{\Omega }\rho s^{s}:\nabla wd\Omega +\int_{S^{C}}\rho c^{s}\cdot wds+\int_{\Omega }\rho w\cdot bd\Omega ,$$

where $S^{s}$ represents the stress tensor of unit mass. $S^{c}$ is the boundary region of stress. Due to the discrete character of MPM, Equation (55) is rewritten as

(56) $$\sum_{p=1}^{N_{p}}m_{p}^{t}[-s^{s}(x_{p}^{t},t):\nabla w{|}_{x_{p}^{t}}+w(x_{p}^{t},t)\cdot c^{s}(x_{p}^{t},t)/h+w(x_{p}^{t},t)\cdot b(x_{p}^{t},t)],$$

The velocity increment of the node is mapped to the material point, and then its update is obtained.

(57) $$dv_{p}^{t}=\sum_{i=1}^{N_{n}}N_{i}(x_{p}^{t})f_{i}^{t}dt/m_{i}^{t},$$

The velocity of the material point at t+dt is calculated as

(58) $$v_{p}^{t+dt}=v_{p}^{t}+dv_{p}^{t},$$

The global coordinate of the material point at this time is

(59) $$x_{p}^{t+dt}=x_{p}^{t}+v_{p}^{t+dt}dt,$$

The strain and Cauchy stress of material point are updated by the strain rate formula, which is defined by

(60) $$\dot{\varepsilon }=[\nabla v+{\left(\nabla v\right)}^{T}]/2,$$

Strain increment:

(61) $$d\varepsilon_{p}^{t}=[\nabla v+{\left(\nabla v\right)}^{T}]dt/2,$$

Strain of material point:

(62) $$\varepsilon_{p}^{t+dt}=\varepsilon_{p}^{t}+d\varepsilon_{p}^{t},$$

**Figure 6:**
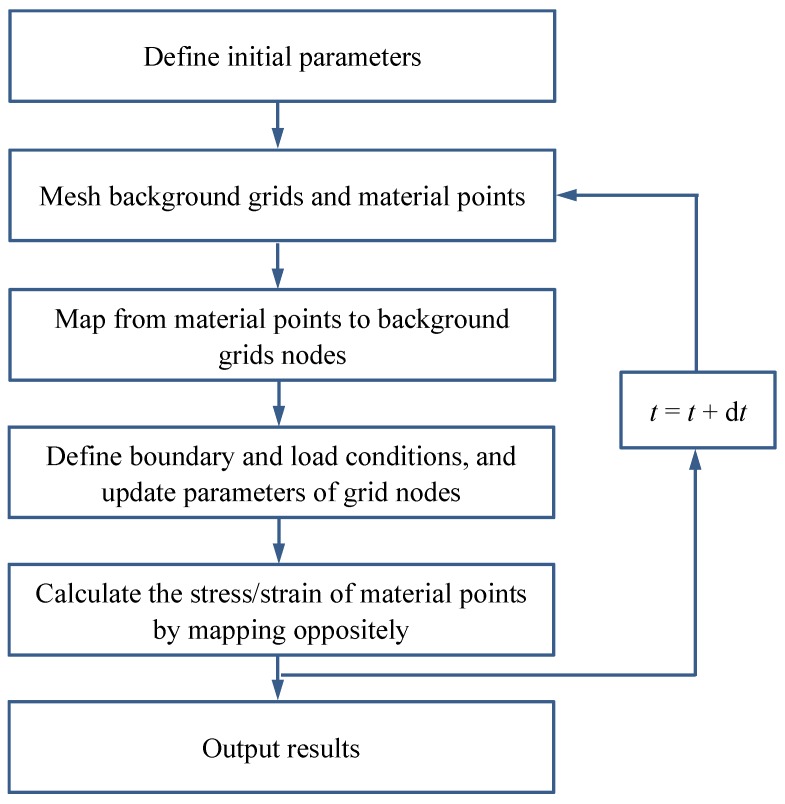
Flow chart of the analysis of MPM.

Then, the Cauchy stress is derived from the constitutive model. The flow chart of material point method is shown in Figure 6.

**Figure 7:**
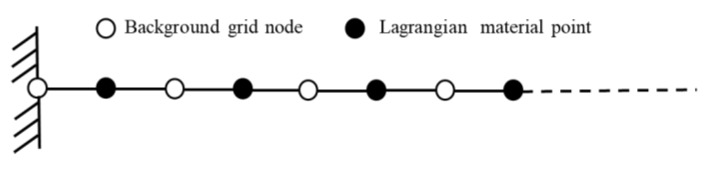
Schematic of background nodes and material points in a one-dimensional model.

Figure 7 shows a one-dimensional rod model, the length of which is 1 m. The left boundary is fixed.

## 3. Results and Discussion

### 3.1. Stress Wave Analysis

The conservation equations for wave propagation mainly include mass and momentum equations.

Mass conservation:

(63) $$\frac{\partial v}{\partial X}=\frac{\partial \varepsilon }{\partial t},$$

Momentum conservation:

(64) $$\rho_{0}\frac{\partial v}{\partial t}=\frac{\partial \sigma }{\partial X},$$

According to the combination of Equations (63) and (64), the velocity of stress wave *C* is derived from

(65) $$C^{2}={\frac{\partial X}{\partial t}\frac{\partial X}{\partial t}|}_{w}=\frac{1}{\rho_{0}}\frac{d\sigma }{d\varepsilon },$$

The substitution of Equation (65) into Equation (64), the following equation is obtained

(66) $$\frac{\partial v}{\partial t}=C^{2}\frac{\partial \varepsilon }{\partial X},$$

Since *ε* and *v* is the first derivative of displacement *u* versus *X* and *t*, Equation (66) is rewritten as

(67) $$\frac{\partial^{2}u}{\partial t^{2}}-C^{2}\frac{\partial^{2}u}{\partial X^{2}}=0,$$

In addition, the compatible relation at the wave front satisfies

(68a) dX=±C  dt,

(68b) dv=±C  dε,

(68c) $$d\sigma =\pm \rho_{0}C\;dv,$$

where *C* is determined as $C=\sqrt{\frac{1}{\rho_{0}}\frac{d\sigma }{d\varepsilon }}$.

**Figure 8:**
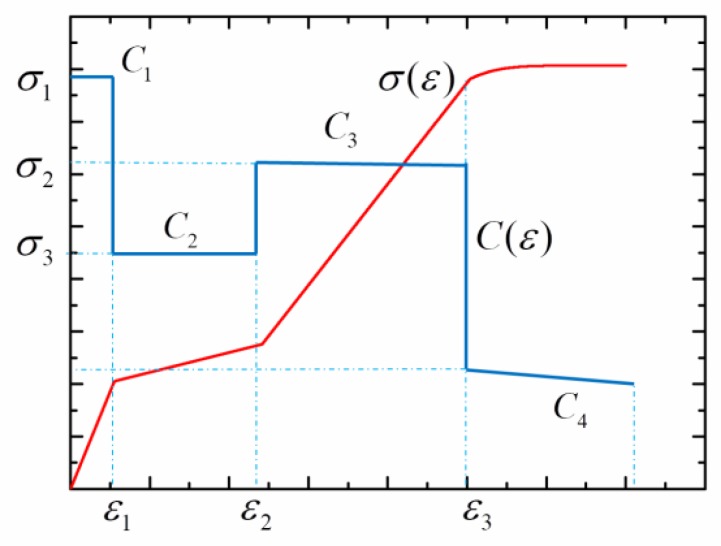
The stress–strain response and its induced stress wave velocity.

During the wave propagation in NiTi alloy rods, *C* is not a constant but a function of the strain *ε*. Such a phenomenon is different from the elastic wave. For a slightness rod with no initial stress, it is seen from Equation (68) that the strain increases by increasing the impact velocity. When *ε* reaches the plastic yield point, there is a three-wave structure in the rod, namely an elastic wave, a phase transition wave, and a plastic wave. Figure 8 exhibits the stress–strain response and the evolution of stress wave velocity.

**Figure 9:**
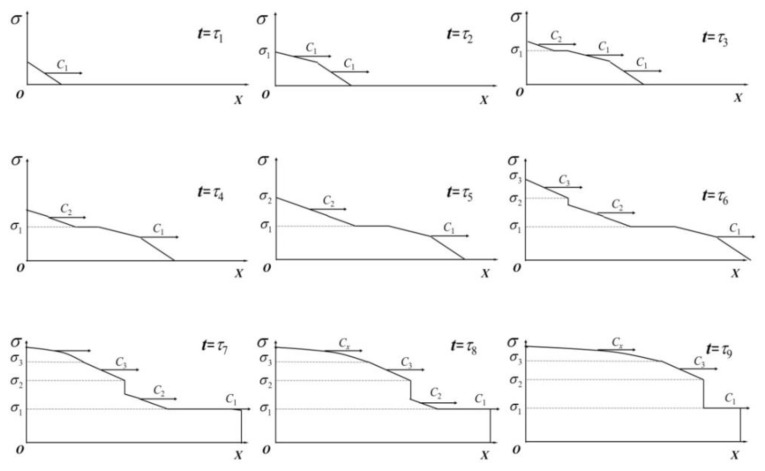
Stress profiles under some instantaneous stages.

Indeed, four stages are always in an order of priority. The austenitic elastic wave appears at first. Subsequently, the phase transition wave occurs, and it is followed by the martensite elastic wave and the plastic wave. Because the martensite elastic wave speed is larger than the one of phase transition wave, the phase transition wave disappears when the martensite elastic wave exceeds the phase transition wave. Figure 9 indicates the stress profiles under some instantaneous stages. While it is just a schematic diagram with no physical meaning, the change in the slope of the stress profile is mainly used to differentiate the waves. From the figure, only the elastic wave propagating in the rod is found at $t=\tau_{1}$ and $t=\tau_{2}$, with a velocity of *C*_1_. When $t=\tau_{3}$, $t=\tau_{4}$, and $t=\tau_{5}$, the phase transition occurs on the boundary, and it propagates with a velocity of *C*_2_. Because the velocity of phase transition wave is less than that of the elastic wave, the distance between these two waves increases gradually. It is also discovered that the rod segment that is located at the critical point of phase transition becomes longer with the wave propagation. After the phase transition, the boundary enters the martensitic elastic stage at $t=\tau_{6}$, and its wave propagates with a velocity of *C*_3_. Similarly, the wave velocity during the phase transition is less than that of the martensitic elastic wave. Thus, the phenomenon of martensitic elastic wave pursuing the phase transition wave is observed by numerical simulations. When $t=\tau_{7}$, $t=\tau_{8}$, and $t=\tau_{9}$, the boundary reaches the plastic deformation stage. It is noteworthy that the elastic wave follows the phase transition wave until the latter disappears. Afterwards, such a three-wave structure degenerates into a double-wave structure, namely, two elastic waves and a plastic wave.

**Figure 10:**
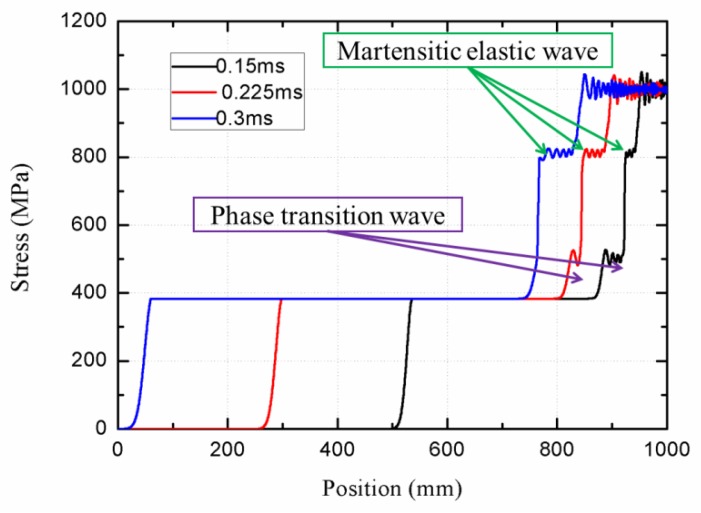
The degeneration of wave structure simulated by MPM.

When the stress loading boundary is set as 500 MPa at first 0.075 ms and then it changes to 1000 MPa, the degeneration behavior of wave structure is observed from MPM simulation, shown in Figure 10. An obvious three-wave structure can be seen at 0.15 ms, and then the martensitic elastic wave pursues the phase transition wave. Finally, the phase transition wave disappears at 0.3 ms. Consequently, the three-wave structure degenerates into a double-wave structure. In addition, the propagation velocities of the stress wave in each phase are obtained. The austenite elastic wave velocity is about 3200 m/s and the phase transition wave velocity is about 640 m/s. The velocity of martensitic elastic wave and plastic wave is 1300 m/s and 800 m/s, respectively. The accuracy of these calculated data is quantified and compared to the predictions by Bekker et al. [20]. They reported that the austenite elastic wave velocity was 3300 m/s. The phase transition wave velocity was about 650 m/s, and the plastic wave was 810 m/s. The difference errors exhibit a reasonable agreement, suggesting the efficiency of our model.

### 3.2. Reflection Analysis

**Figure 11:**
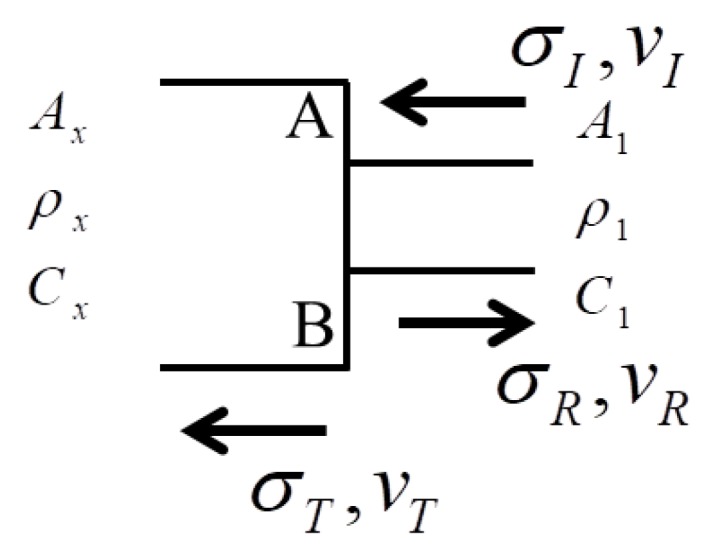
Schematic of wave reflection and transmission.

Figure 11 shows the wave reflection and transmission. $\sigma_{I}$ and $\nu_{I}$ are the stress and particle velocity of incident wave, respectively. $\sigma_{R}$ and $\nu_{R}$ are the stress and particle velocity of reflection wave, respectively. $\sigma_{T}$ and $\nu_{T}$ are the stress and particle velocity of transmission wave, respectively. Indeed, these variables could be solved by mass and momentum conservation equations.

The balance of force on the surface AB is given by

(69) $$A_{1}(\sigma_{I}+\sigma_{R})=A_{x}\sigma_{T},$$

The conservations of mass and momentum on the surface AB imply:

(70a) $$v_{I}-v_{R}=v_{T},$$

(70b) σAdt=ρAdxv,

The substitution of Equation (70b) into Equation (70a) produces

(71) $$\left(\sigma_{I}-\sigma_{R})/(\rho_{1}c_{1})=\sigma_{T}/(\rho_{x}c_{x}\right),$$

$\sigma_{T}$ and $\sigma_{R}$ are achieved through Equations (69) and (71).

(72a) $$\sigma_{T}=\frac{2A_{1}\rho_{x}c_{x}}{A_{x}\rho_{x}c_{x}+A_{1}\rho_{1}c_{1}}\sigma_{I},$$

(72b) $$\sigma_{R}=\frac{A_{x}\rho_{x}c_{x}-A_{1}\rho_{1}c_{1}}{A_{x}\rho_{x}c_{x}+A_{1}\rho_{1}c_{1}}\sigma_{I},$$

$\sigma_{R}\to \sigma_{I}$ is obtained when $A_{x}\to \infty$ and, $\sigma_{T}\to 0$. This suggests that the stress of reflection wave is identical to the one of incident wave when the boundary is fixed. Therefore, the total stress is increased to two times compared to the original one.

**Figure 12:**
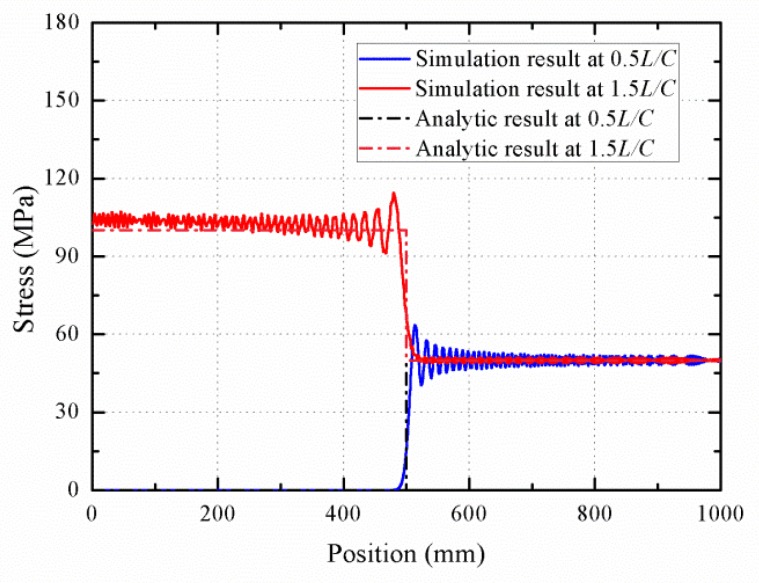
Stress profiles during the propagation and reflection of austenite elastic wave.

To verify the accuracy of the present theory and demonstrate its capability of prediction, Figure 12 shows the propagation and reflection of elastic wave in austenite phase. For comparison, $t=0.5L/C_{1}$ and $t=1.5L/C_{1}$ are chosen to exhibit the stress profile in the rod. As shown in the figure, the wave propagation and reflection of austenite elastic stage is identical to that of the general elastic wave, exhibiting no phase transformation. The stress wave velocity with *C*_1_ is increased by two times after it reflects from the fixed boundary. The difference errors indicate that the proposed model seems to be reasonable for the description of stress wave propagation and reflection.

**Figure 13:**
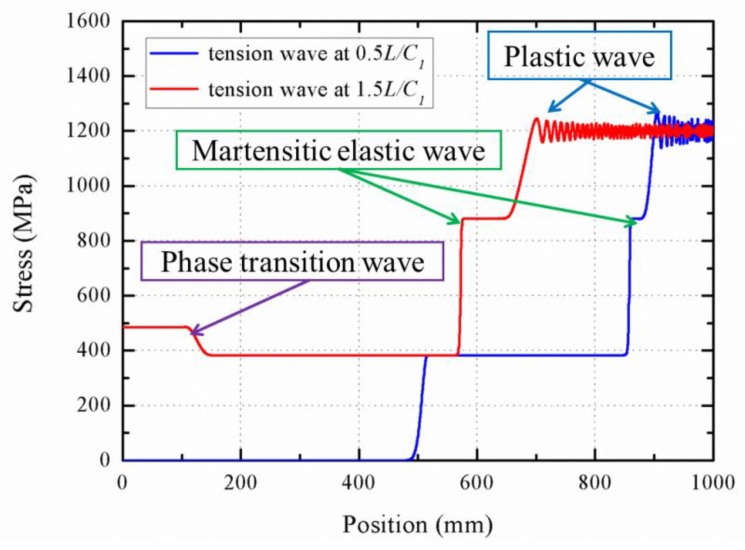
Stress profiles during the propagation and reflection of stress wave under high stress level.

When the load exceeds the plastic yield point, the three-wave structure appears in the bar, which is different from the elastic wave. Figure 13 illustrates the stress profile in high stress conditions. It is found that the velocity of austenite elastic wave propagation is the fastest one among those three waves. Accordingly, the rod segment that stays at the critical point of phase transition becomes longer during the wave propagation. This phenomenon is consistent with the conclusion of the above theoretical results.

However, when the austenite elastic wave is reflected at the fixed boundary, the stress increases by a different way compared with the elastic one. The phase transition seems to occur in the reflection process, which could be explained that the reflected wave produces an increment of the stress over the critical point. Thus, Equation (71) is rewritten as the following form

(73) $$\frac{\sigma_{I}}{\rho_{1}C_{1}}-\frac{\sigma_{R}}{\rho_{1}C_{2}}=\frac{\sigma_{T}}{\rho_{x}C_{x}},$$

where *C*_1_ is the velocity of incident wave. *C*_2_ is the velocity of reflected wave, and it is also the velocity of phase transformation wave.

$\sigma_{R}$ is obtained by solving Equations (69) and (73),

(74) $$\sigma_{R}=\frac{C_{2}}{C_{1}}\frac{A_{x}\rho_{x}C_{x}-A_{1}\rho_{1}C_{1}}{A_{1}\rho_{1}C_{2}+A_{x}\rho_{x}C_{x}}\sigma_{I},$$

$\sigma_{R}\to \frac{C_{2}}{C_{1}}\sigma_{I}$ is found when $A_{x}\to \infty$. Therefore, the stress increment is not $\sigma_{I}$, but $\frac{C_{2}}{C_{1}}\sigma_{I}$. Furthermore, Figure 13 shows that the reflection wave propagate goes back with the velocity of phase transition wave, which is only twenty percent of the elastic wave velocity with about 3200 m/s. Besides, it is found that the plastic wave velocity is similar to that of the phase transformation wave, by comparing the distance of the wave propagation in the same period, which is proved by the fact that the two waves propagate about 120 mm at the time step with $0.5L/C_{1}$. While the velocity of martensitic elastic wave is larger than that of phase transformation, the phase transition wave disappears in the initial stage of wave propagation. With the propagations of martensitic elastic wave and the plastic one, the rod segment that stays at the plastic yield point is expanded.

### 3.3. Effect of the Loading Direction

**Figure 14:**
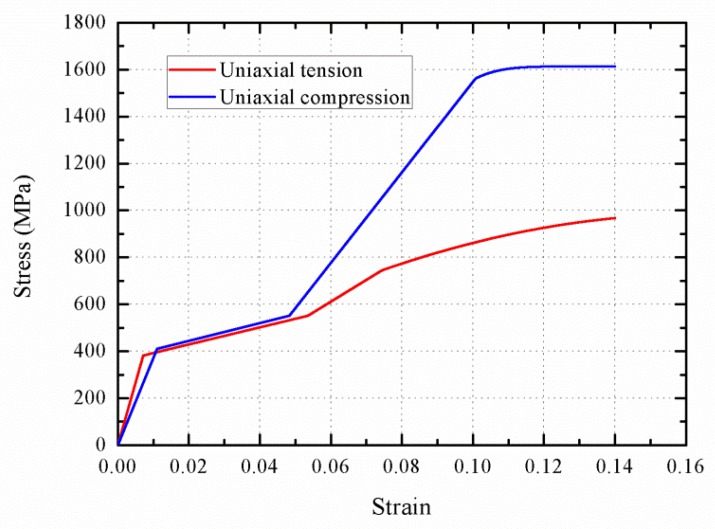
The stress–strain response with various loading directions.

**Figure 15:**
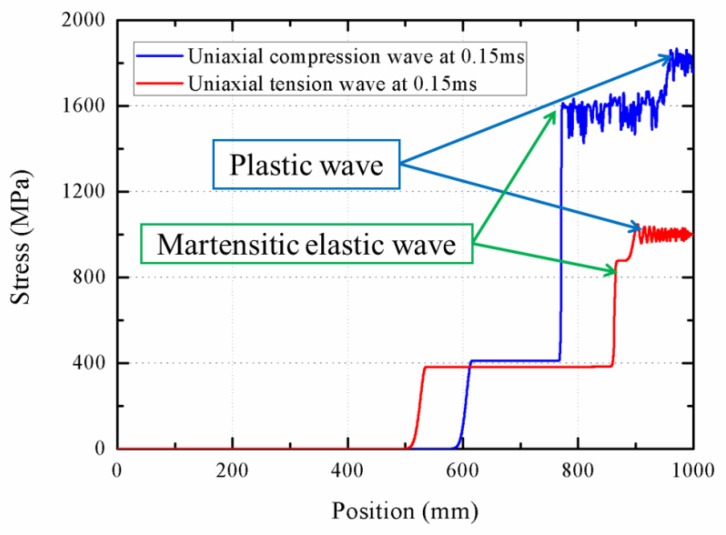
Stress profiles during the wave propagation with various loading directions.

To investigate the influence of loading direction on wave propagation, Figure 14 and Figure 15 show the stress–strain curve and the stress profile of wave propagation at 0.15 ms with various loading directions, respectively. There are two differences between the compression and tension loading. For the martensitic elastic stage, the strain range from 0.05 to 0.1 for the compression phase is longer than the one between 0.055 and 0.07 for the tension phase. In addition, the martensitic elasticity compression modulus is larger than that under tension. However, Figure 15 shows that the austenite elastic and plastic wave propagate faster under tension loading, compared to the compression loading. This is attributed to the fact that the austenite elastic and plastic compression stiffness is less than the tension one. The plastic wave velocity during the tension loading process is predicted as about 800 m/s, which is two times than that of the compression plastic wave. Due to the difference in the austenite elastic wave velocity with different loading directions, the rod segment that stays at the start point of phase transition is longer under tension loading. Similarly, the rod segment that stays at the start point of plastic is also longer during compression loading, which is derived from the difference in martensitic elastic waves.

### 3.4. Effect of the Strain Rate

**Figure 16:**
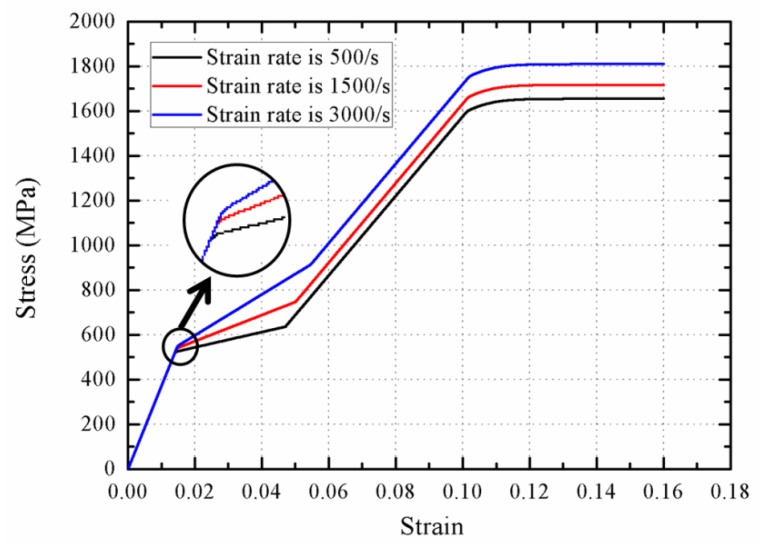
The stress–strain response with various strain rates.

**Figure 17:**
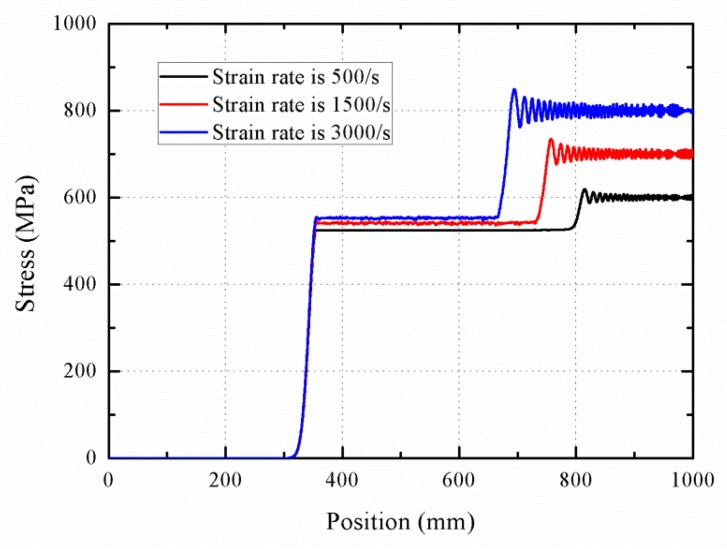
Stress profiles during the wave propagation with various strain rates.

Equation (21) is used to describe the phase transition stress. Figure 16 shows the stress–strain curve with various strain rates, and in turn the stress profiles of wave propagation at 0.25 ms are provided in Figure 17. With increasing the strain rate, the initial phase transition strain increases by a slight manner. In contrast, the increase of final phase transition strain is significant, and the strain increases approximately from 0.05 at 500/s to 0.055 at 3000/s. It is evident that the stiffness of phase transition stage increases with the increasing strain rate. The wave velocity during the phase transition increase from 800 to 1200 m/s, indicating that the rod segment that stays at the initial point of phase transition becomes shorter with increasing the strain rate.

## 4. Conclusions

In this study, a dynamic analysis is presented under the framework of MPM, with a special focus on the propagation of stress waves in a NiTi rod under one-dimensional uniaxial loading.

(1) It is found that the stress wave obtained exhibits an obvious three-wave structure. During the process of wave propagation, the wave velocity in the phase transition is less than the one of austenite elastic wave, and the martensitic elastic wave follows the phase transition wave till the phase transition wave disappear. Herein, the three-wave structure degenerates into a double-wave structure.
(2) Due to the occurrence of the phase transition wave during the wave reflection at the fixed boundary, the stress does not increase to two times as much as that of the original one. The stress increment is proportional to *C*_2_, which is proved by MPM solution.
(3) The influences of loading direction and stain rate are investigated comprehensively. It is found that the velocity of phase transition wave increases with increasing strain rate. In addition, the loading direction has a distinct effect on the martensitic elastic wave and plastic wave propagation. The martensite elastic wave velocity under tensile condition is less than that with compressive loading, but the plastic wave velocity under compression is less than that by tensile loading.

## Figures and Tables

**Table 1:** Chemical compositions of NiTi alloys.

Ni	Co	Cu	Cr	Fe	Nb	C	H	O	N	Ti
55.72%	0.005%	0.005%	0.005%	0.012%	0.005%	0.045%	0.001%	0.03%	0.001%	44.17%

**Table 2:** Material parameters of NiTi alloys under tensile loading.

Parameter	Value	Unit	Parameter	Value	Unit
$E_{A0}$	53,453	MPa	$E_{M0}$	9280	MPa
$\nu_{A}$	0.3	-	$\nu_{M}$	0.3	-
$\sigma_{s0}^{tr}$	380	MPa	$\sigma_{f0}^{tr}$	552	MPa
$\sigma_{y0}^{p}$	730	MPa	$\varepsilon_{m}$	0.044	-
*C*_A_	3.7	MPa/K	*C_M_*	5.4	MPa/K
*C*_1_	0.038	-	*C*_2_	0.075	-
*C*_3_	0.012	-			
*k*_1_	400	-	*k*_2_	600	-
*k*_3_	400	-	*k*_4_	800	-
*z*_1_	10	MPa	*z*_2_	170	MPa
*n*_1_	3.0	-	*n*_2_	3.0	-
*m*	50	-	*R*_1_	260	MPa

**Table 3:** Material parameters of NiTi alloys under compressive loading.

Parameter	Value	Unit	Parameter	Value	Unit
$E_{A0}$	37,130	MPa	$E_{M0}$	19,280	MPa
$\nu_{A}$	0.3	-	$\nu_{M}$	0.3	-
$\sigma_{s0}^{tr}$	409	MPa	$\sigma_{f0}^{tr}$	552	MPa
$\sigma_{y0}^{p}$	1550	MPa	$\varepsilon_{m}$	0.034	-
*C*_A_	-	MPa/K	*C_M_*	5.4	MPa/K
*C*_1_	0.0337	-	*C*_2_	0.0002	-
*C*_3_	4 × 10^−5^	-			
*k*_1_	400	-	*k*_2_	800	-
*k*_3_	400	-	*k*_4_	800	-
*z*_1_	10	MPa	*z*_2_	170	MPa
*n*_1_	3.0	-	*n*_2_	3.0	-
*m*	250	-	*R*_1_	50	MPa
